# Host Antiviral Response Suppresses Ciliogenesis and Motile Ciliary Functions in the Nasal Epithelium

**DOI:** 10.3389/fcell.2020.581340

**Published:** 2020-12-21

**Authors:** Qianmin Chen, Kai Sen Tan, Jing Liu, Hsiao Hui Ong, Suizi Zhou, Hongming Huang, Hailing Chen, Yew Kwang Ong, Mark Thong, Vincent T. Chow, Qianhui Qiu, De-Yun Wang

**Affiliations:** ^1^Department of Otolaryngology, Zhujiang Hospital, Southern Medical University, Guangzhou, China; ^2^Department of Otolaryngology, Infectious Diseases Translational Research Programme, Yong Loo Lin School of Medicine, National University of Singapore, Singapore, Singapore; ^3^Department of Otolaryngology, Head and Neck Surgery, Guangdong Provincial People’s Hospital, Guangdong Academy of Medical Science, Guangzhou, China; ^4^Department of Otolaryngology, Head and Neck Surgery, National University Health System, National University Hospital, Singapore, Singapore; ^5^Infectious Diseases Translational Research Programme, Department of Microbiology and Immunology, Yong Loo Lin School of Medicine, National University Health System, National University of Singapore, Singapore, Singapore

**Keywords:** respiratory virus, antiviral response, human nasal epithelial cells (hNECs), cilia, mucociliary barrier

## Abstract

**Background:**

Respiratory viral infections are one of the main drivers of development and exacerbation for chronic airway inflammatory diseases. Increased viral susceptibility and impaired mucociliary clearance are often associated with chronic airway inflammatory diseases and served as risk factors of exacerbations. However, the links between viral susceptibility, viral clearance, and impaired mucociliary functions are unclear. Therefore, the objective of this study is to provide the insights into the effects of improper clearance of respiratory viruses from the epithelium following infection, and their resulting persistent activation of antiviral response, on mucociliary functions.

**Methods:**

In order to investigate the effects of persistent antiviral responses triggered by viral components from improper clearance on cilia formation and function, we established an *in vitro* air–liquid interface (ALI) culture of human nasal epithelial cells (hNECs) and used Poly(I:C) as a surrogate of viral components to simulate their effects toward re-epithelization and mucociliary functions of the nasal epithelium following damages from a viral infection.

**Results:**

Through previous and current viral infection expression data, we found that respiratory viral infection of hNECs downregulated motile cilia gene expression. We then further tested the effects of antiviral response activation on the differentiation of hNECs using Poly(I:C) stimulation on differentiating human nasal epithelial stem/progenitor cells (hNESPCs). Using this model, we observed reduced ciliated cell differentiation compared to goblet cells, reduced protein and mRNA in ciliogenesis-associated markers, and increased mis-assembly and mis-localization of ciliary protein DNAH5 following treatment with 25 μg/ml Poly(I:C) in differentiating hNECs. Additionally, the cilia length and ciliary beat frequency (CBF) were also decreased, which suggest impairment of ciliary function as well.

**Conclusion:**

Our results suggest that the impairments of ciliogenesis and ciliary function in hNECs may be triggered by specific expression of host antiviral response genes during re-epithelization of the nasal epithelium following viral infection. This event may in turn drive the development and exacerbation of chronic airway inflammatory diseases.

## Introduction

The airway epithelial cells represent the primary line of defense against foreign materials entering the airway. This is accomplished via mechanical means of mucus secretion and removal of the material facilitated by rhythmic beating of the motile cilia (Gudis et al., 2012), as well as immune responses that further remove pathogenic infections (Yan et al., 2016; Tan et al., 2019). However, in chronic airway inflammatory diseases, impairment of ciliary architecture and function often accompanies aberrant immune responses against foreign materials (Thomas et al., 2010; Liu et al., 2018). Such impairment may contribute to a vicious cycle of increased susceptibility to pathogens and repeated chronic inflammations (Gudis et al., 2012; Fokkens et al., 2020).

Respiratory viral infections are implicated as drivers and exacerbators of chronic airway inflammatory diseases (Tan et al., 2017, 2020). Respiratory viruses such as respiratory syncytial virus (RSV) directly affect the ciliary architecture and function (Smith et al., 2014), while influenza virus (IFV) and rhinovirus (RV) infections lead to the breakdown of the epithelial barrier (Tan et al., 2020). During such infections, the viruses first infect and damage the epithelial cells, where loss of the epithelial barrier occurs as a result of direct viral-induced cell death, or removal of infected cells by cell-mediated immune responses. After an infection is cleared, the epithelium will need to undergo re-epithelization through the expansion and differentiation of the airway basal cells to repair and reform the barrier damaged by the virus. However, patients with chronic airway inflammatory diseases often have reduced effectiveness in their antiviral immunity (Akbarshahi et al., 2018; Ravi et al., 2019). This results in viruses or viral components continuing to linger in the airway, constantly triggering pathogen sensors and antiviral immunity (Marin et al., 2000; Kling et al., 2005; Wood et al., 2011). Such constant activation of antiviral immune response can potentially affect the re-epithelization following infection, thus driving the pathogenesis and exacerbation of chronic airway inflammatory diseases (Cho et al., 2013; Ravi et al., 2019). Currently, the understanding of ciliary impairment and reduction in chronic airway inflammatory diseases leans toward the expression of Th type 2-biased inflammatory cytokines such as IL-4, IL-5, and IL-13 (Grosse-Onnebrink et al., 2016; Liu et al., 2018; Huang et al., 2020). On the other hand, the influence of Th type 1-biased inflammatory cytokines (which are expressed in response to virus infection) on cilia formation, assembly, and function remains understudied.

We have previously reported impaired and aberrant ciliogenesis and ciliary assembly in chronically inflamed airways (Peng et al., 2018, 2019a; Qiu et al., 2018). Furthermore, from recent transcriptomics study of chronic rhinosinusitis with nasal polyp (CRSwNP) cohorts in the Asian region, we observed that enrichment of antiviral immune response genes accompanies the abnormalities in ciliogenesis and assembly (Peng et al., 2019b). Finally, reports of *in vitro* infection of human nasal epithelial cells (hNECs) with IFV, which is known to trigger strong interferon-induced antiviral responses, also showed reduced expression of cilia component genes such as dynein axonemal heavy chain 5 (*DNAH5*) and dynein axonemal assembly Factor 5 (*DNAAF5*), as well as ciliogenesis genes such as multiciliate differentiation and DNA synthesis-associated cell cycle protein and cyclin O (*MCIDAS* and *CCNO*) (Boon et al., 2014; Qiu et al., 2018; Tan et al., 2019). By analyzing these findings from the literature, it is therefore suggested that viral infection, specifically the antiviral responses it induces, may contribute to the impairment of cilia formation and assembly in the airway, thereby driving and aggravating chronic inflammation in the airways.

Therefore, we hypothesized that incomplete clearance of respiratory viruses in an inflammed airway post-infection may constantly trigger antiviral responses. This event then drives the development and exacerbation of chronic airway inflammatory diseases via suppressed ciliogenesis and ciliary assembly, thus favoring goblet cell differentiation that manifests as symptoms of chronic airway inflammatory diseases such as CRSwNP and asthma (Marin et al., 2000; Wood et al., 2011; Ravi et al., 2019). To test this hypothesis, we established an *in vitro* air-liquid interface (ALI) cell culture system of hNECs and provided constant antiviral stimulation using a viral surrogate Poly(I:C) to mimic remnants of viral components (viral RNA) during re-epithelization. The objective of this study is therefore to investigate the effects of constant antiviral response activation due to incomplete viral clearance toward the motile ciliary formation and assembly in the nasal epithelium during re-epithelization following infection. This will enable us to establish the association between long-term effects of impaired viral clearance and ciliary impairment in perpetuating the symptoms of chronic airway inflammatory diseases (Bryche et al., 2019; Menzel et al., 2019).

## Materials and Methods

### Human Nasal Epithelial Cells (hNECs) and Their Differentiation in Air-Liquid Interface (ALI) Cultures

Approval to conduct this study was obtained from the National Healthcare Group Domain-Specific Board of Singapore (DSRB Ref: D/11/228) and institutional review board of the National University of Singapore (IRB Ref: 13-509). Healthy nasal epithelial tissues were obtained from patients undergoing nasal septum correction surgery at the Department of Otolaryngology, Head and Neck Surgery, National University Hospital, Singapore. There was no airway inflammation at the point of collection; and antihistamine, glucocorticoid, or other anti-inflammatory drugs were not used from 1 month before surgery. In addition, no other systemic diseases were found in the donors. Written informed consents were obtained from the patients prior to the operation. Primary epithelial cells were isolated from the freshly collected nasal inferior turbinate mucosa, then cultured in our human nasal epithelial stem/progenitor cells (hNESPCs) culture system according to previously standardized protocol (Zhao et al., 2012). Briefly, primary cells were cultured in and expanded with Dulbecco’s Modified Eagle Medium: Nutrient Mixture F-12 (DMEM/F12) (Gibco-Invitrogen, Carlsbad, CA, United States) containing 0.1 nM of Cholera toxin (Sigma, St. Louis, MO, United States), 5 μg/mL of Insulin human (Sigma, St. Louis, MO, United States), 10 ng/mL of Human Epithelial Growth Factor (EGF, Gibco-Invitrogen, Carlsbad, CA, United States), 0.5 μg/mL of Hydrocortisone Stock Solution (STEMCELL Technologies Inc., Vancouver, Canada), 2 nM/mL of 3,3′,5-triiodo-l-thyronine (T3) (Sigma, St. Louis, MO, United States), 100 IU/mL of Antibiotic-antimycotic solution (Gibco-Invitrogen, Carlsbad, CA, United States), and 10 μL/mL of N-2 supplement (Gibco-Invitrogen, Carlsbad, CA, United States) with a 3T3 feeder layer to select and enrich for hNESPCs (Li et al., 2014). The hNESPCs were then transferred into a 24-well 0.4 μm Transwell inserts (Corning, NY, United States) for differentiation to hNECs. Once the cells were attached to the well, growth medium was discarded and 350 μL of PneumaCult^TM^ -ALI Medium with inducer supplements (STEMCELL Technologies Inc., Vancouver, Canada) was added to the basal chamber for ALI conditions. Then the cells were cultured for 3–4 weeks, with medium change every 2–3 days, to form pseudostratified layers of hNECs mimicking the nasal epithelium for subsequent experiments. Details of the ALI culture procedure can be found from our previous study (Li et al., 2014). Each n number used for the experiments represented hNECs cultured from an individual donor. Details of donor information could be found in Supplementary Table S1.

### Inoculation of Influenza (H3N2, H1N1, B/Victoria) and Rhinovirus in Fully Differentiated Human Nasal Epithelial Cells (hNECs)

The virus strains and multiplicity of infection (MOI) used in this study were as follows: Human Influenza H3N2 Virus was of the strain A/Aichi/2/1968, MOI 0.1 (ATCC, Manassas, VA, United States). Human Influenza H1N1 Virus was of the strain Singapore/G2/25.1/2014, MOI 0.1. Human Influenza B/Victoria lineage (B/Vic) was of the strain B/Singapore/G2-14.1/2014, MOI 0.1. Human Rhinovirus (HRV) used in this study is HRV A16, MOI 2.5 (Qiao et al., 2018; Tan et al., 2018; Tian et al., 2018; Chen et al., 2019; Ivan et al., 2020).

Prior to infection, fully differentiated hNECs were washed with 1 × Dulbecco’s phosphate-buffered saline (dPBS) and infected with each virus at their designated MOI then incubated for 1 h at 35°C. After the incubation, the viral inoculum was removed and the hNECs were incubated back in 35°C for their respective time points. The control hNECs were harvested for RNA prior to the infection at 0 h post-infection (hpi). The infected hNECs were then harvested for RNA at 24 and 48 hpi.

### Stimulation of Human Nasal Epithelial Cells (hNECs) Culture With Poly(I:C)

In a separate setup, hNESPCs were transferred to transwells, allowed to grow to confluency before transitioning to ALI culture for differentiation in PneumaCult^TM^-ALI Medium. At the start of the ALI culture, cells grown in medium for control, or medium containing 5, 10, 25, 50, and 100 μg/ml low MW (400–1250 bp) Polyinosinic-polycytidylic acid sodium salt (Poly[I:C]) (Sigma, St. Louis, MO, United States) in the basal chamber to simulate re-epithelization of nasal epithelium under antiviral state activation. The Poly(I:C) treatment was replenished together since the cells started ALI-culture, with the medium changed every 2–3 days. Subsequently, the concentration of 25 μg/ml was selected for the actual re-epithelization (RE) setup experiments. During the 3 weeks of ALI culture where cells differentiated into hNECs, cells were monitored for their TEER, cell count, ciliary beat frequency (CBF), gene and protein expression analysis at days 7, 14, and 21 post-ALI.

For treatment of poly(I:C) on fully differentiated (FD) hNECs setup, the medium containing 25 μg/ml low MW (400–1,250 bp) poly(I:C) was added from day 21 post-ALI and replenished with every medium change every 2–3 days. After 7- or 14-day posttreatment, cells were monitored for their cell count, ciliary beating frequency (CBF), gene and protein expression analysis.

### RNA Extraction and Real-Time Quantitative PCR

Total RNA was extracted from the hNECs using the mirVana miRNA isolation kit (Life Technologies, Grand Island, NY, United States). The extracted RNA was then quantified by nanodrop analysis, and 1 μg of total RNA was subjected to cDNA synthesis using Maxima Reverse Transcriptase kit (Thermo Scientific, Pittsburgh PA, United States) according to the manufacturer’s protocol. Then, qPCR analysis was performed to evaluate the transcriptional levels of genes selected based on previous RNA-seq and whole-transcriptome sequencing analysis using pre-designed primers (Sigma, St. Louis, MO, United States). Each qPCR reaction was performed in duplicate using GoTaq-qPCR Master Mix kit (Promega, San Luis Obispo, CA, United States). Relative gene expression was calculated using the comparative 2^−ΔΔCt^ method normalized against the housekeeping gene PGK1 (viral inoculation experiment) or RPL13A [Poly(I:C) stimulation], respectively. Details of primer sequences could be found in Supplementary Table S2.

### Cytospin Preparation and Immunofluorescence Staining

At each collection, single-cell suspensions (1–2 × 10^5^ cells) were dissociated from transwells using 0.5 × Trypsin/EDTA solution (Gibco, Carlsbad, CA, United States) at 37°C. The dissociated cells were then fixed in 4% paraformaldehyde at room temperature for 10 min, followed by two washes with 1 × dPBS and pelleted. Last, cytospin (1–2 × 10^4^ cells per slide) preparations were prepared at 500 rpm for 5 min with mild acceleration using Shandon Cytospin 4 Cytocentrifuge (Thermo Fischer Scientific, United States).

Before incubation with the primary antibody (overnight at 4°C) and secondary antibody (1 h at room temperature), the cytospin slides were permeabilized with 0.1% Triton X-100 and blocked with 10% Goat Serum (Invitrogen). Rabbit polyclonal anti-DNAH5 (1:500, ab122391) and mouse monoclonal antibody directed against acetylated alpha tubulin (1:800, ab24610) were obtained from Abcam. Rabbit polyclonal anti-Foxj1 (1:300, HPA005714), anti-MCIDAS (1:100, HPA058073), anti-CCNO (1:50, HPA050090), and, anti-ZO1 (1:300, HPA001636) were obtained from Sigma. Rabbit polyclonal anti-MUC5AC (1:500, sc-21701) was obtained from Santa Cruz. Highly cross absorbed secondary antibodies, including Alexa Fluor -488 to mouse (1:500, A11029) and -594 to rabbit (1:500, A11037) were purchased from Invitrogen. Immunofluorescence images were taken with a fluorescent microscope (Olympus IX51, Tokyo, Japan).

For transwell membrane IF staining, the wells were fixed directly with 4% paraformaldehyde, then similarly permeabilized and stained as per the procedure staining cytospin slides.

### Semiquantitative Dynein Axonemal Heavy Chain 5 (DNAH5) Grading System in Cytospin IF Staining

Three patterns of DNAH5 localization are defined, including Normal (presence throughout the axoneme), Partial (undetectable in the distal part of the axoneme), and Absence (completely missing throughout the entire axoneme). We developed a semiquantitative scoring system for DNAH5 based on our previous study for which Normal (scored 1), Partial (scored 2), and Absence (scored 3), then compared the average score between groups (Qiu et al., 2018).

### Live Cell Counting

Trypan Blue Solution was used to assess the cell number of hNECs post-exposure to Poly(I:C). It was performed with cell count on the hemocytometer after dissociating the hNECs from the transwells. Only live cells, i.e., cells that did not take up the blue dye were counted.

### Measurement of Cilia Length, Ciliated and Goblet Cell Percentage by IF Staining

Cilia length was evaluated in hNECs isolated from day 21 post-ALI culture by assessing alpha-tubulin staining at × 400 magnification. Five areas of cilia staining from cytospin slides were randomly selected for each group and compared between the control and Poly(I:C)-treated group. Cilia length was measured with ImageJ software. The mean values of cilia length were calculated from 20 measurements of each area.

The percentage of ciliated and goblet cells from cytospin samples (hNECs from ALI culture) were counted using the following formula:

$$\frac{\text{Number}\text{of}\text{alpha}\text{tubulin}\text{positive}\text{cells}\mathrm{or}\mathrm{MUC5AC}\text{positive}\text{cells}}{\text{Total}\text{cell}\text{number}\left(\mathrm{DAPI}\mathrm{staining}\right)}$$

### Ciliary Beat Frequency Assessment

Prior to measurement, cells were washed with 37°C 1 × dPBS. CBF was then analyzed at room temperature by using the Sisson-Ammons Video Analysis system (SAVA, Omaha, NE, United States) with an inverted microscope (Olympus, Tokyo, Japan) at a magnification of 400×. Actively beating ciliated cells from each experiment in ALI culture were assessed, respectively, before harvesting. For all experiments, the predominant frequency of a small group of cilia from each sample was viewed and taken in at least five separate fields every 30 s for up to 3 min, while they were maintained at a constant temperature (23 ± 0.5°C). All frequencies from each sample are expressed as the mean from each field over 3 min (Li et al., 2014).

### *Trans*-Epithelial Electrical Resistance Measurement

To determine if the integrity of airway epithelial is disrupted after Poly(I:C) treatment, we performed trans-epithelial electrical resistance (TEER) measurement, using an epithelial volt/ohm meter (EVOM2) (World Precision Instruments, United States), following the manufacturer’s instruction. In each well of controls and Poly(I:C) experiments, TEER was measured at ALI day 14 and day 21. The value of TEER was calculated by subtracting the blank well measurement (Li et al., 2014) and presented as Ω⋅cm^2^.

### Statistical Analysis

Statistical analysis was performed using GraphPad Prism 8 software (GraphPad Software Inc., San Diego, CA, United States). Data are presented as median ± interquartile range (IQR) unless otherwise stated. Mann–Whitney test was used to compare the mean values of different treatment groups unless otherwise stated. *P* < 0.05 was considered statistically significant.

## Results

### Human Influenza Virus and Rhinovirus Infections Reduce Expression of Ciliated Genes in Human Nasal Epithelial Cells

**FIGURE 1:**
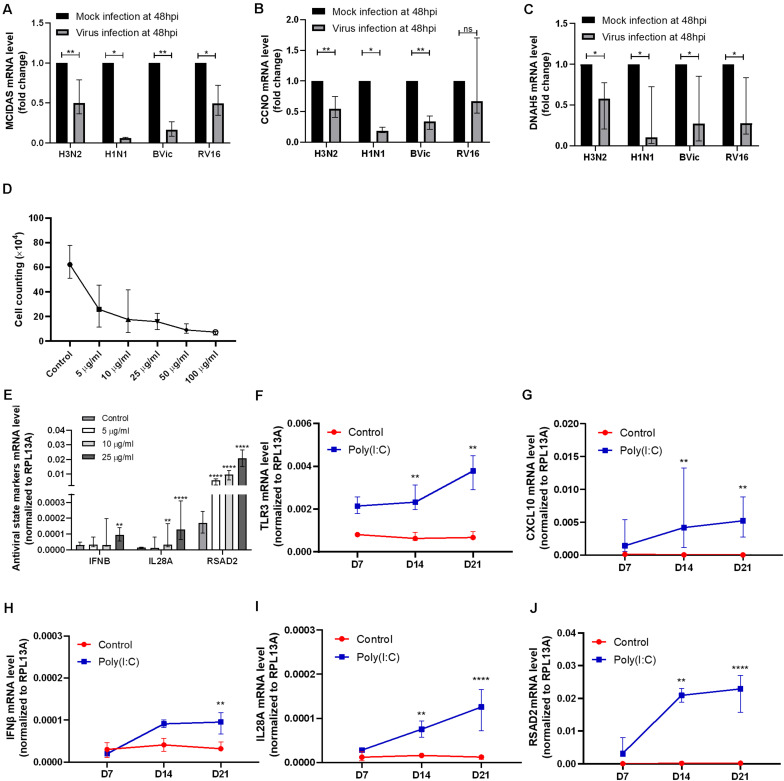
Confirmation of cilia marker reduction in viral infection and antiviral response genes induction in polyinosinic-polycytidylic acid and sodium salt [Poly(I:C)] treatment. **(A–C)** *MCIDAS* mRNA level was decreased in H3N2, H1N1, B/Vic, and RV16 infections (*P* = 0.0078, *P* = 0.0156, *P* = 0.0078, *P* = 0.0156, *n* = 8, respectively) at 48 hpi in human nasal epithelial cells (hNECs). Cell cycle protein and cyclin O (*CCNO)* mRNA level was decreased in H3N2, H1N1, and B/Victoria lineage (B/Vic) infections (*P* = 0.0078, *P* = 0.0156, *P* = 0.0078, *n* = 8, respectively) at 48 hpi in hNECs. Dynein axonemal heavy chain 5 (*DNAH5)* mRNA level was decreased with H3N2, H1N1, B/Vic and RV16 infections (*P* = 0.0156, *P* = 0.0234, *P* = 0.0156, *P* = 0.0234, *n* = 8, respectively.) at 48 hpi in hNECs. **(D)** Live cell counting of hNECs treated by Poly(I:C) in air–liquid interface (ALI) culture on day 21 of ALI culture. *X* axis indicates the concentrations of Poly (I:C) (μg/ml) used. **(E–J)** Antiviral state markers type I interferon (*IFNB*), type III interferon (*IL28A*), and interferon-stimulated gene (*RSAD2*) mRNA level was increased significantly post 25 μg/ml of Poly(I:C) treatment (data was compared to control, *n* = 9). *TLR3* and *CXCL10* were also activated by 25 μg/ml of Poly(I:C) treatment during hNEC differentiating (*P* < 0.05 is presented as “^∗^.” P < 0.01 is presented as “^∗∗^.” P < 0.0001 is presented as “^****^.” Ns, not significant. ALI—D7, *n* = 3; D14, *n* = 6; D21, *n* = 9. Analysis was performed with Mann–Whitney test. Plot: median with interquartile range (IQR).

As shown in our previous study, we observed a negative correlation between antiviral state activation and ciliary gene expression in CRSwNP patients (Peng et al., 2019b). To verify the association, we first compared gene expression changes in viral infection of hNECs. From infection studies, we found enriched interferon genes, antiviral genes, and abnormal expression of ciliated cell-associated markers after H3N2 influenza virus infection (Supplementary Figure S1) (Tan et al., 2019). We observed increased *IFNB1*, *IL29*/*IFNL1*, *IL28A*/*IFNL2*, *IFNL3*, *RSAD2*, and *CXCL10* post infection. These antiviral genes are also observed in infections with other respiratory viruses such as RV and RSV (Huong et al., 2018; Tan et al., 2018). This is accompanied by reduction in ciliogenesis markers, *MCIDAS* and *CCNO*, and increase in *OFD1*, a gene that encodes a centrosomal protein inhibiting ciliogenesis (Wang et al., 2016). In addition, cilia dynein markers *DNAH5* and *DNAAF5* were found to be reduced, whereas *TUBB6* was elevated following IFV H3N2 infection. The H3N2 transcriptome findings were further confirmed using qPCR in other virus strains including IFV (H1N1, H3N2, and B/Vic), and human rhinovirus (HRV A16). Interestingly, *DNAH5*, *MCIDAS*, and *CCNO* exhibited consistent descending trends in all infections tested, suggesting this observation to be applicable to multiple respiratory viruses (Figures 1A–C). Following this confirmation, we next investigated if the observation persisted post infection, which is the main aim of our study. However, as active viral infection results in substantial cell death over time (Yan et al., 2016; Tian et al., 2018), and without viral clearance mechanisms in an *in vitro* system, the decrease may be due to more infected cells dying over time. Therefore, to minimize the effect of viral infection and to investigate the effects of constant antiviral state activation after viral clearance on ciliary formation and assembly in hNECs, we treated the hNECs using Poly(I:C) to simulate viral components post infection. This will enable us to assess if the reduction of ciliary mRNAs is independent of cell death due to direct infection, thus serving as a surrogate of re-epithelization in an *in vitro* system, where viruses cannot be cleared.

### Repeated Dosing of 25 μg/ml Poly(I:C) Simulates a Constant Antiviral State in Human Nasal Epithelial Cells

Poly(I:C) treatment on hNECs was started at the initiation of ALI-culture to simulate the effect of antiviral state activation on growth and differentiation of hNECs. At day 21, we observed that the total number of cells were reduced following treatment with any concentration of Poly(I:C), potentially due to cell death or inhibition of cell division (Figure 1D). On the other hand, antiviral state markers *IFNB*, *IL28A* (*IFNL2*), and *RSAD2* increased most significantly with 25 μg/ml of Poly(I:C) treatment (Figure 1E). Given that all treatments reduced the number of cells, the concentration of 25 μg/ml was selected for the subsequent antiviral state experiments in hNECs due to the potency of this concentration in activating antiviral state.

### Constant Antiviral State Activation by Poly(I:C) Results in Inhibition of Human Nasal Epithelial Cell Differentiation and Impairment of Ciliated Cell Formation, Assembly, and Function

By using Poly(I:C) to mimic viral component persistence post viral clearance, we showed that the constant treatment of Poly(I:C) activated the antiviral state as seen by the increase in viral sensor *TLR3*, interferon *IFNB* and *IL28A*, interferon-stimulated gene *RSAD2*, and antiviral cytokine *CXCL10* (Figures 1F–J). The activation of antiviral state in turn altered the formation and functions of multiple components in the mucociliary barrier, which may impact the re-epithelization (RE).

#### Poly(I:C) Treatment Resulted in Aberrant Differentiation of Human Nasal Epithelial Cells Into Multi-Ciliated Epithelium

**FIGURE 2:**
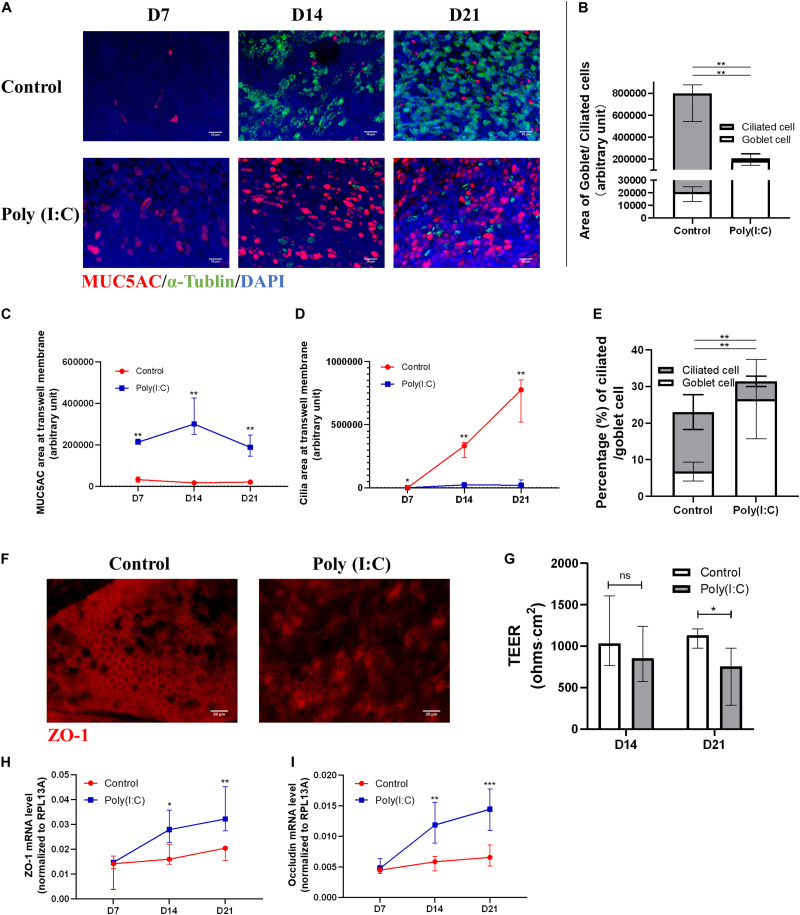
Antiviral state activation affects ciliated cell differentiation and tight junction formation. **(A–D)** Representative transwell IF images of goblet (MUC5AC) and ciliated (alpha-tubulin) cells double staining at ALI day 7/14/21 (×200 magnification, scale bar = 10 μm), in which MUC5AC and cilia area were measured with five views of the same donor. **(E)** Percentage of ciliated cell/goblet cell counting from cytospin at ALI day 21, respectively (counted at × 200 magnification, *n* = 5). **(F)** Representative transwell IF images of tight junction marker (ZO-1) at ALI day 21 (×400 magnification, scale bar = 20 μm). **(G)** Trans-epithelial electrical resistance (TEER) of Poly(I:C) group was significantly reduced at ALI day 21 (*P* = 0.0379, *n* = 8) compared with control, while showing a reducing trend at ALI day 14 (*n* = 8). The total TEER (ohms⋅cm^2^) is presented by TEER measurement (ohms) × Area of a membrane (cm^2^). **(H,I)** mRNA levels of tight junctions markers *ZO-1* and *Occludin* both increased significantly at ALI day 14 and 21. (*P* < 0.05 is presented as “^∗^.” *P* < 0.01 is presented as “^∗∗^.” *P* < 0.001 is presented as “^∗∗∗^.” ALI—D7, *n* = 3; D14, *n* = 6; D21, *n* = 9). Analysis was performed using Mann–Whitney test. Plot: median with IQR.

We observed that, over time, with transwell staining, hNESPCs in ALI differentiated into hNECs following different courses of differentiation in control and Poly(I:C)-treated group (Figure 2A). While both groups achieved near complete differentiation at day 21 post-ALI, the control group differentiated normally with a larger ratio of area of ciliated cell (α-Tubulin+) to goblet cell (MUC5AC+), in stark contrast with Poly(I:C) group, showing very low area ratio of ciliated to goblet cells (Figures 2B–D). In the cytospin staining of ciliated versus goblet cells, we similarly observed a smaller percentage of ciliated cells (4.88% ± 1.45% vs. 16.24% ± 4.72%), but more goblet cells (MUC5AC-positive cells, 26.57% ± 10.82% vs. 6.78% ± 2.55%) in Poly(I:C) treatment group compared to control (Figure 2E). The similarity between transwell stain and cytospin stain indicate that cytospin cells are reflective of the differentiation and development on the transwell. In terms of mRNA expression, *MUC5AC* mRNA was significantly elevated at day 21 post-ALI in Poly(I:C)-treated group (Supplementary Figure S2A), while no significant difference was observed for expression of *MUC5B*, *TUBB*, and *p63* (Supplementary Figures S2B–D). *KRT5* mRNA was significantly decreased at day 14 post-ALI in the Poly(I:C)-treated group, which coincided with large decrease in *Ki67* expression in the Poly(I:C)-treated group (Supplementary Figures S2E–F). The reduction of *Ki67* may indicate reduction in cell division, suggesting that the reduction of cell number following Poly(I:C) treatment may be partially attributed to inhibition of cell division. In addition, the tight junction marker ZO-1 was reduced in the Poly(I:C)-treated group, which affected tight junction formation and subsequently the TEER, especially at day 21 post-ALI (Figures 2F,G). Interestingly, in response to the compromised tight junction, *ZO-1* and *Occludin* mRNA level were elevated, potentially as an attempt to restore the tight junction (Figures 2H,I). These results indicate that an antiviral state induced by Poly(I:C) compromised the ability of hNESPCs to differentiate into a multiciliated epithelium, which may represent an impediment during the re-epithelization of the hNECs following viral damage and improper viral clearance.

#### Reduced Ciliogenesis Markers During Human Nasal Epithelial Cell Differentiation Under Antiviral State

**FIGURE 3:**
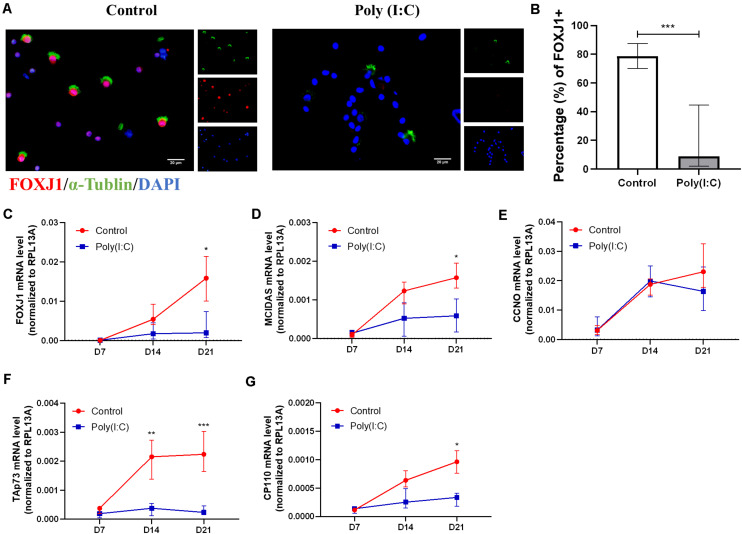
Antiviral state activation reduced ciliogenesis marker in differentiating hNECs. **(A)** Representative cytospin IF images of ciliogenesis marker FOXJ1 (×400 magnification, scale bar = 20 μm). **(B)** Percentage of FOXJ1 (ALI day 21) nuclear positive staining in total cell count was significantly decreased post Poly(I:C) treatment. (counted at × 200 magnification, *P* = 0.0006, *n* = 8). **(C–G)** mRNA levels of ciliogenesis-associated markers *FOXJ1*, *MCIDAS*, *TAp73, CP110*, but not CCNO were significantly downregulated at ALI day 21 in the Poly(I:C) group. (*P* < 0.05 is presented as “^∗^.” *P* < 0.01 is presented as “^∗∗^.” *P* < 0.001 is presented as “^∗∗∗^.” ALI—D7, *n* = 3; D14, *n* = 6; D21, *n* = 9.). Analysis was performed using Mann–Whitney test. Plot: median with interquartile IQR.

Following the observation of aberrant formation of hNECs, we next assessed the ciliogenesis marker expression and distribution following Poly(I:C) treatment. Among the ciliogenesis markers, FOXJ1 showed the greatest difference between Poly(I:C)-treated group and control. At day 21 post-ALI, we found that the percentage of FOXJ1-positive cells decreased significantly (*P* = 0.0006, *n* = 6) in Poly(I:C) groups, and FOXJ1 staining were absent even in ciliated cells of the treatment group (Figures 3A,B). This large difference coincided with the day showing the largest FOXJ1 mRNA increase as well (Figure 3C). Similarly, the percentages of MCIDAS, CCNO, Tap73, and CP110 mRNAs also remained low in Poly(I:C)-treated cells (Figures 3D–G), while no difference was observed in OFD1 mRNA (Supplementary Figure S2G). In contrast to the mRNA, however, MCIDAS and CCNO-positive staining was not significant between the groups over time, although the Poly(I:C) group exhibited a reduced trend for both markers (Supplementary Figure S3). Hence, our finding suggested the reduction of ciliated cells during differentiation with Poly(I:C) (i.e., re-epithelization under improper viral clearance) may arise due to the reduction in ciliogenesis markers that shunted the differentiation toward goblet cell formation.

#### Impairment of Ciliary Architecture, Assembly, and Function in Human Nasal Epithelial Cells Under Antiviral State Activation

**FIGURE 4:**
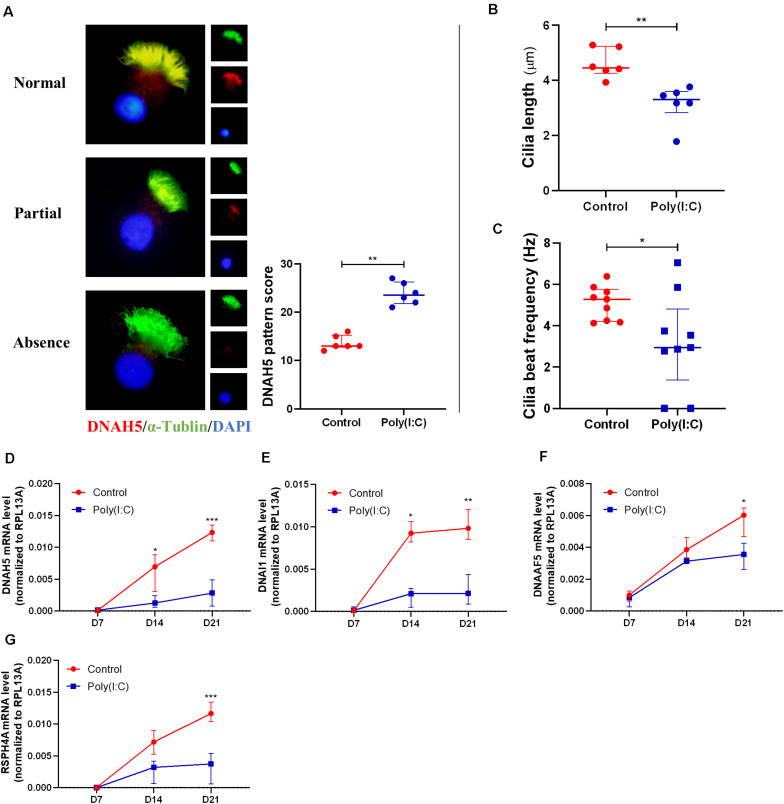
Antiviral state activation induced aberrant cilia assembly and functions. **(A)** Representative cytospin IF images of DNAH5 (× 1,000 magnification). Sample staining showing three patterns of DNAH5 localization—Normal expression, Partial expression, and Absence of DNAH5. A semiquantitative DNAH5 grading system in cytospin from hNECs scored the patterns Normal, Partial, and Absence at 1, 2, and 3, respectively. Scoring of DNAH5 localization showed that absence or mislocalization of DNAH5 was more common in the Poly(I:C) group (*P* = 0.0022, *n* = 6, ALI day 21). **(B)** Cilia length of hNECs at ALI day 21 in cytospin preparation measured by ImageJ at ×400 magnification (*P* = 0.0022, *n* = 6, ALI day 21). **(C)** Cilia beat frequency (CBF) was auto-analyzed using SAVA system at ALI day 21. CBF from two donor samples were undetectable following Poly(I:C) treatment (*P* = 0.0306, *n* = 9, ALI day 21); as the control have normal cilia, these samples were therefore included in the analysis as they remained classified as cilia producing phenotype. **(D–G)** mRNA level of cilium assembly markers. *DNAH5*, *DNAI1*, *DNAAF5*, and *RSPH4A* reduced significantly at ALI day 21 in Poly(I:C) group. (*P* < 0.05 is presented as “^∗^.” *P* < 0.01 is presented as “^∗∗^.” *P* < 0.001 is presented as “^∗∗∗^.” ALI—D7, *n* = 3; D14, *n* = 6; D21, *n* = 9.). Analysis was performed using the Mann–Whitney test. Plot: median with IQR.

Upon establishing that Poly(I:C) treatment as a surrogate of improper viral clearance during re-epithelization affects the differentiation of hNECs, we next investigated the ciliated cells formed from the differentiation under both conditions. In single hNECs cytospin sections, we were able to assess the cilia assembly of differentiated ciliated cells. We achieved this by evaluating the distribution and localization of DNAH5, a dynein protein responsible for ciliary movement, on the ciliary axoneme. Under normal circumstances, the entire axoneme of the cilia in a healthy ciliated cell will express DNAH5, whereas in inflamed tissues and cells, the expression of DNAH5 is limited to the basal area of the axoneme, or absent entirely (Figure 4A). The localization was previously adapted into a scoring system to assess defects in ciliary architecture and assembly (Qiu et al., 2018). In the Poly(I:C)-treated group, we found that the localization score was increased, indicating defective cilia architecture and assembly (Figure 4A). In agreement with this, cilia length from the control groups and Poly(I:C) groups were 4.62 ± 0.53 and 3.15 ± 0.71 μm, respectively at ALI-culture day 21. The results showed a 32% decrease in cilia length in cells treated with Poly(I:C) (Figure 4B). The defects of cilia architecture and length in turn affected ciliary functions, where we observed significant reductions in CBF measurements (Figure 4C). At the same time, we also observed that the mRNA level of *DNAH5* and another three representative cilia markers *DNAI1*, *RSPH4A*, and *DNAAF5* remained consistently lower in the Poly(I:C)-treated group (Figures 4D–G). These data further suggest that antiviral state activation not only reduced ciliated cell differentiation, but also affected the remaining cilia structurally and functionally.

#### Treatment of Poly(I:C) on Fully Differentiated Human Nasal Epithelial Cells Mainly Affects Ciliated to Goblet Cell Ratio

To assess if Poly(I:C) treatment affected fully differentiated hNECs as well, we treated the 21-day post-ALI hNECs similarly for 7 and 14 days. In response to the Poly(I:C) treatment, cell numbers were also reduced over time but not to the extent of the RE setup, indicating that both cell division inhibition and cell death played a role in the cell number reduction of the RE experiments (Supplementary Figure S4). Overall, the treatment of Poly(I:C) in the treatment of fully differentiated cells resulted in lesser impact on the cells, potentially due to the cells being terminally differentiated with their cilia fully developed. However, we did observe an increasing trend in goblet cell numbers following treatment (Supplementary Figure S5). Poly(I:C) treatment also do not significantly affect cilia architecture and CBF, even though there were increasing trends in antiviral responses following treatment (Supplementary Figures S6–S8). Goblet cell and tight junction markers mRNA remained unchanged while ciliogenesis marker *MCIDAS*, *Tap73*, *CP110*, and *FOXJ1*; and ciliary marker *DNAH5*, *DNAI1*, *DNAAF5*, and *RSPH4A* trended toward reduction in the Poly(I:C)-treated group, but not achieving the significant reduction in actual live virus infection (Supplementary Figures S9–S11). This lower impact of Poly(I:C) on fully differentiated cells may potentially be attributed to the intact epithelium being able to effectively remove the surrogate of viral remnants, whereas in a re-epithelizing epithelium, the differentiated cells are absent, and the effects on the basal cells may strongly affect the differentiation into normal hNECs.

## Discussion

Respiratory viral infection is one of the major factors of chronic airway inflammatory disease development and exacerbation, due to the pathogens causing the impairment of epithelium, affecting epithelial repair and worsening the symptoms of existing disease (Hashimoto et al., 2008; Viniol and Vogelmeier, 2018). Among various respiratory viruses, influenza virus and rhinovirus are largely associated with the development, progression, and exacerbation of chronic airway inflammatory diseases (Costa et al., 2014; Oliver et al., 2014; Jartti and Gern, 2017; Zheng et al., 2018). During the replication process, cell host defenses are activated via the release of antiviral factors and cytokines to combat the infection (Busse et al., 2010). Generally, infected airway epithelial cells release type I interferons (IFNα/β), type III interferons (IFNλ), interferon effectors, and adaptive immunity signaling cytokines and chemokines as the infection progresses (Wark and Gibson, 2006; Singh et al., 2010). These responses serve to clear the viruses from the airway, but also inflict direct and indirect destruction to the epithelial barrier. Additionally, respiratory viruses also possess mechanisms to evade the immune responses to prolong their infections. Viral infections such as RV and RSV can promote a Th type 2-biased inflammation, which can drive asthma or CRSwNP development and exacerbations (Becker, 2006; Jurak et al., 2018; Linden et al., 2019). The compromise mucociliary function may also contribute to repeated infections that further modify antiviral responses, resulting in a vicious cycle that impedes the ability to properly clear the viruses. This usually results in remnants of viruses and its components to persist in the airway (Essaidi-Laziosi et al., 2018; Ravi et al., 2019) and may lead to disruption of re-epithelization of the airway following repeated insults. Therefore, it is vital to investigate the role of long-term aberrant antiviral responses in compromising the epithelial barrier, driving the pathogenesis and exacerbation of chronic airway inflammatory diseases.

Our study has identified epithelium-initiated host antiviral responses as contributors to the abnormal mucociliary functions, commonly found in patients with chronic airway inflammatory diseases. This finding is significant as we identified a key event (i.e., antiviral state activation) that triggered mucociliary dysfunction down to the ciliary level in the nasal epithelium. In addition, our study also highlighted the effective use of a relevant human model to unravel the mechanisms of mucociliary alteration *in vitro*. In patients with chronic airway inflammatory diseases, there exist a large number of respiratory viral infection studies that showed the host responses using similar models of hNECs. However, there are very few equivalent studies on the long-term effects of viral component persistence in the airway (Marin et al., 2000; Wood et al., 2011; Ravi et al., 2019). Hence, once we confirmed the factors affected during active viral infection, we further investigated the effects of viral component persistence in activating the antiviral state using Poly(I:C) treatment on hNECs, especially during the repair and re-epithelization of the airway epithelium post infection (Lewandowska-Polak et al., 2018; Ramezanpour et al., 2018). The objective of this study is to offer insights into the long-term effects of respiratory virus infection, by linking persistent antiviral state activation due to viral components to the impairment of mucociliary regeneration, architecture, and functions in the upper airway.

In our previous study on the whole transcriptome in chronic rhinosinusitis with nasal polyps (CRSwNP) with Asian cohorts, we observed an interesting association that the tissues from CRSwNP patients were enriched for antiviral responses, despite not showing active infection (Peng et al., 2019b). Pathways such as the type I IFN signaling pathway and defense response to virus were found to be significantly upregulated. At the same time, ciliary markers were found to be significantly altered in CRSwNP patients. In addition, other studies further showed that cilia markers were mis-localized in CRSwNP tissues, which reverted to normal when grown in an ALI culture (Peng et al., 2018; Qiu et al., 2018). Hence, it is imperative to investigate the exact mechanisms that contribute to the mis-localization, which may culminate in the pathology and exacerbation of chronic airway inflammatory diseases. Incidentally, RNA sequencing of hNECs post-H3N2 influenza virus infection also revealed strong antiviral responses (Tan et al., 2019). At the same time, ciliary markers such as DNAH5, DNAAF5, MCIDAS, and CCNO were downregulated following infection of hNECs (Tang et al., 2013; Horani et al., 2018; Tan et al., 2019; Terre et al., 2019). Therefore, the link between antiviral state activation and ciliary architecture alteration was further explored in this study.

In order to eliminate the confounding effects of an active infection and focus solely on the effects of antiviral activation on mucociliary functions, we exploited a simulated model of *in vitro* re-epithelizing hNECs using Poly(I:C) to mimic viral components for the antiviral state activation. Poly(I:C) (25 μg/ml) successfully stimulated hNEC antiviral defense responses based on the increased mRNA levels of IFNβ, IL28A, and RSAD2. Following up with hNECs cultured with 25 μg/ml of Poly(I:C), our data revealed that the proliferation of hNECs and differentiation to multiciliated epithelium were significantly inhibited and shunted toward goblet cell differentiation (Figure 2). This is in line with clinical observations of reduced cilia formation and mucus hypersecretion observed in chronic upper airway inflammatory diseases such as CRSwNP (Liu et al., 2018; Peng et al., 2019b). In addition, we also observed reduced ciliogenesis (e.g., Foxj1, MCIDAS, and CCNO) in hNECs under antiviral state, which were reduced in both ciliated and non-ciliated cells, suggesting inhibition of both ciliogenesis and proper cilia formation (Figure 3). In addition, we also observed decreased cilia length and CBF, and reduced expression of cilia markers. From the comparison of marker expression patterns via mRNA and IF staining, we showed that ciliogenesis was inhibited as early as day 14 and become more apparent at day 21 post-ALI, while shunting of differentiation toward goblet cells occur from day 7 post-ALI. Finally, we have successfully elucidated that antiviral state activation and its effects are the factors that drive the mislocalization of ciliary proteins in CRSwNP and potentially other chronic airway inflammatory diseases (Figure 4) (Chen et al., 2018; Qiu et al., 2018). Overall, the findings from our study highlighted the significance of the antiviral state genes that were activated following active infections due to improper viral clearance in the upper airway, where the viral components potentiate aberrant re-epithelization of the airway. This then serves as a mechanism to impair the epithelial formation, cilia coverage, motile cilia function, and tight junction barriers, further driving the development and exacerbation of chronic airway inflammatory diseases. Moreover, by identifying the triggers for ciliary protein mislocalization and impairment, different methods to scavenge and neutralize the viral component triggers can be explored to potentially repair and recover the mucociliary functions.

The study is not without its limitations. First, while Poly(I:C) serves to mimic viral components, it only captures the double-stranded RNA components of the activation. Future studies with viral RNA or proteins, recombinant interferons, or inactivated viruses can also be investigated to see if they yield similar or different outcomes toward ciliary impairment during re-epithelization. Further, other factors such as metabolic and energy changes to the cells following Poly(I:C) stimulation that may contribute to ciliary impairment were not explored in this study. These can be part of the future exploration that would further pinpoint the other contributory factors that lead to the impairment of mucociliary functions.

## Conclusion

**FIGURE 5:**
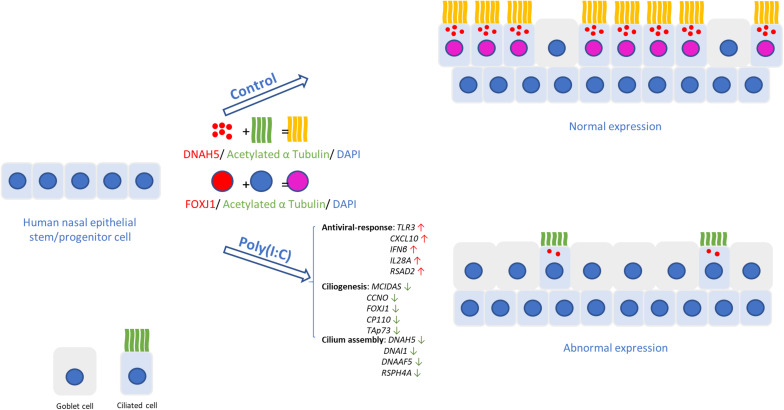
Changes in human nasal epithelial cell caused by host antiviral response. In hNECs differentiating while antiviral state is active due to improper viral clearance, fewer ciliated cells with shorter cilia were observed. The induction of interferons from viral components induced goblet cell formation and reduced expression of ciliogenesis and cilium assembly markers, causing reduced ciliated cell and impairment of the remaining ciliated cells, thereby compromising cilia function and mucociliary barrier.

Taken together, we investigated the underlying mechanisms that lead to impairments in ciliogenesis and ciliary assembly and ciliary function in hNECs, induced by antiviral state activation using simulated viral components (Figure 5). The cause of these pathologic changes is likely intrinsic to the mechanism of host defense based on the *in vitro* measurements of ciliary function (e.g., DNAH5, RSPH4A, and DNAAF5) and ciliogenesis-associated markers (e.g., FOXJ1, MCIDAS, CP110, and TAp73). These findings are of vital importance for identifying the driving mechanisms that contribute to the development and exacerbation of chronic upper airway inflammatory diseases such as CRSwNP, which have high risk of recurrence following treatment. A better understanding of the link found between the antiviral state and ciliary impairment during reepithelization will facilitate the development of more specific treatments for restoring the mucociliary functions, in order to improve the management of chronic upper airway inflammatory diseases.

## Data Availability Statement

The raw data supporting the conclusions of this article will be made available by the authors, without undue reservation.

## Ethics Statement

Approval to conduct this study was obtained from the National Healthcare Group Domain Specific Board of Singapore (DSRB Ref: D/11/228) and institutional review board of the National University of Singapore (IRB Ref: 13-509). Written informed consent was obtained from the patients.

## Author Contributions

QC, KT, VC, QQ, and D-YW designed and carried out the experiment and analyzed the data. JL and HO supervised the *in vitro* cell culture study and participated in the experiments. SZ, HH, and HC participated in the data analysis. MT and YO provided the samples. QC and KT contributed equally to this work. All the authors have read and agreed to the published version of the manuscript.

## Conflict of Interest

The authors declare that the research was conducted in the absence of any commercial or financial relationships that could be construed as a potential conflict of interest.

## Supplementary Material

Supplementary Figure 1 Heat map of log fold changes for defense response to virus compared to mock infected control. Ciliogenesis and cilia-assembly markers were differentially expressed at 24 and 48 h post-H3N2 infection, which accompanies induction of antiviral response genes. The color red denotes up-regulation, blue denotes down-regulation, while gray denotes that the gene was not significantly differentially expressed.

Supplementary Figure 2 **(A,B)** mRNA level of goblet cell markers *MUC5AC* and *MUC5B*. **(C,G)** mRNA level of cilia-associated markers *TUBB* and *OFD1*. **(D–F)** mRNA level of stem cell and proliferation markers *p63*, *KRT5*, and *Ki-67*. Analysis was performed using Mann–Whitney test. Plot: median with IQR. (*P* < 0.05 are presented as ‘^∗^.’ *P* < 0.01 are presented as ‘^∗∗^.’ ns, not significant. ALI – D7, *n* = 3; D14, *n* = 6; D21, *n* = 9.

Supplementary Figure 3 **(A)** Representative cytospin IF images of ciliogenesis marker MCIDAS in differentiating hNECs at ALI – D14 and D21 (×200 magnification, scale bar = 10 μm). **(B)** Percentage of MCIDAS nuclear positive staining in total cells (counted in × 200 magnification, ALI – D14, *n* = 3; D21, *n* = 3.). **(C)** Representative cytospin IF images of ciliogenesis marker CCNO in differentiating hNECs at ALI – D14 and D21 (×200 magnification, scale bar = 10 μm). **(D)** Percentage of CCNO nuclear positive staining in total cells (counted in × 200 magnification, ALI – D14, *n* = 3; D21, *n* = 3.). Data was analyzed using Mann–Whitney test. Plot: median with IQR.

Supplementary Figure 4 Live cell count of fully differentiated hNECs treated by Poly(I:C) in ALI culture for 7 days and 14 days. (FD: fully differentiated). Data was analyzed using Mann–Whitney test. Plot: median with IQR.

Supplementary Figure 5 Representative pictures of MUC5AC (red) and α-Tubulin (green)’s cytospin IF staining in fully differentiated hNECs post Poly(I:C) treatment for 7 and 14 days. Data showed the percentages of goblet and ciliated cell counted in cytospin (counted in × 200 magnification, *n* = 3). (FD: fully differentiated). Plot: median with IQR.

Supplementary Figure 6 DNAH5 pattern score in fully differentiated hNECs post Poly(I:C) treatment for 7 and 14 days. (FD: fully differentiated. ALI – FD-D7: *n* = 3; FD-D14: *n* = 3). Plot: median with IQR.

Supplementary Figure 7 CBF in fully differentiated hNECs post Poly(I:C) treatment for 7 and 14 days. (FD: fully differentiated. ALI – FD-D7: *n* = 3; FD-D14: *n* = 3). Data was analyzed using Mann–Whitney test. Plot: median with IQR.

Supplementary Figure 8 **(A–E)** mRNA level of antiviral response markers in fully differentiated hNECs post Poly(I:C) treatment for 7 and 14 days. (FD: fully differentiated. ns, not significant. ALI – FD-D7: *n* = 3; FD-D14: *n* = 3). Data was analyzed using Mann–Whitney test. Plot: median with IQR.

Supplementary Figure 9 **(A–C)** mRNA level of *MUC5AC* and TJ markers (*ZO-1* and *Occludin*) in fully differentiated hNECs post Poly(I:C) treatment for 7 and 14 days. (FD: fully differentiated. ns: not significant. ALI – FD-D7: *n* = 3; FD-D14: *n* = 3). Data was analyzed using Mann-Whitney test. Plot: median with IQR.

Supplementary Figure 10 **(A–E)** mRNA level ciliogenesis markers in fully differentiated hNECs post Poly(I:C) treatment for 7 and 14 days. (FD, fully differentiated. ns, not significant. ALI – FD-D7: *n* = 3; FD-D14: *n* = 3). Data was analyzed using Mann–Whitney test. Plot: median with IQR.

Supplementary Figure 11 **(A–D)** mRNA level cilium assembly markers in fully differentiated hNECs post Poly(I:C) treatment for 7 and 14 days. (FD, fully differentiated. ns, not significant. ALI – FD-D7: *n* = 3; FD-D14: *n* = 3). Data was analyzed using Mann–Whitney test. Plot: median with IQR.

Supplementary Table 1 Donor information of hNECs sources.

Supplementary Table 2 Primer sequences used in RT-qPCR.
