# Supplementary material for: Host Antiviral Response Suppresses Ciliogenesis and Motile Ciliary Functions in the Nasal Epithelium
Source: Front Cell Dev Biol. 2020 Dec 21;8:581340. doi: 10.3389/fcell.2020.581340 (PMC7779769; doi:10.3389/fcell.2020.581340)
Supplement: Supplementary Figure 1 — Heat map of log fold changes for defense response to virus compared to mock infected control. Ciliogenesis and cilia-assembly markers were differentially expressed at 24 and 48 h post-H3N2 infection, which accompanies induction of antiviral response genes. The color red denotes up-regulation, blue denotes down-regulation, while gray denotes that the gene was not significantly differentially expressed. [file Data_Sheet_1.docx]

Supplementary Material

# Supplementary Figures and Tables

## Supplementary Figures


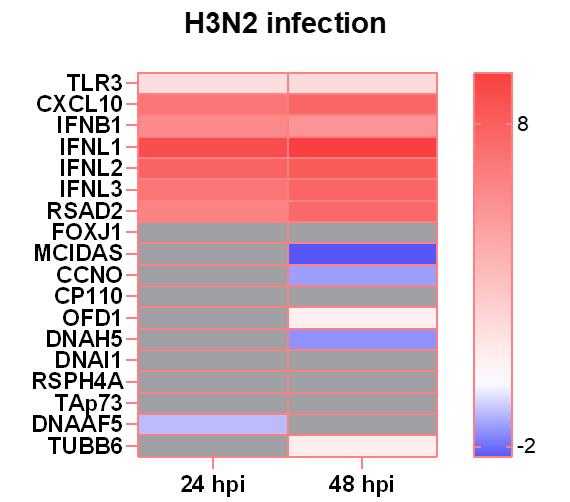


**Supplementary Figure 1.** Heat map of log fold changes for defense response to virus compared to mock infected control. Ciliogenesis and cilia-assembly markers were differentially expressed at 24 hpi and 48 hpi post-H3N2 infection, which accompanies induction of antiviral response genes. The color red denotes up-regulation, blue denotes down-regulation, while grey denotes that the gene was not significantly differentially expressed.


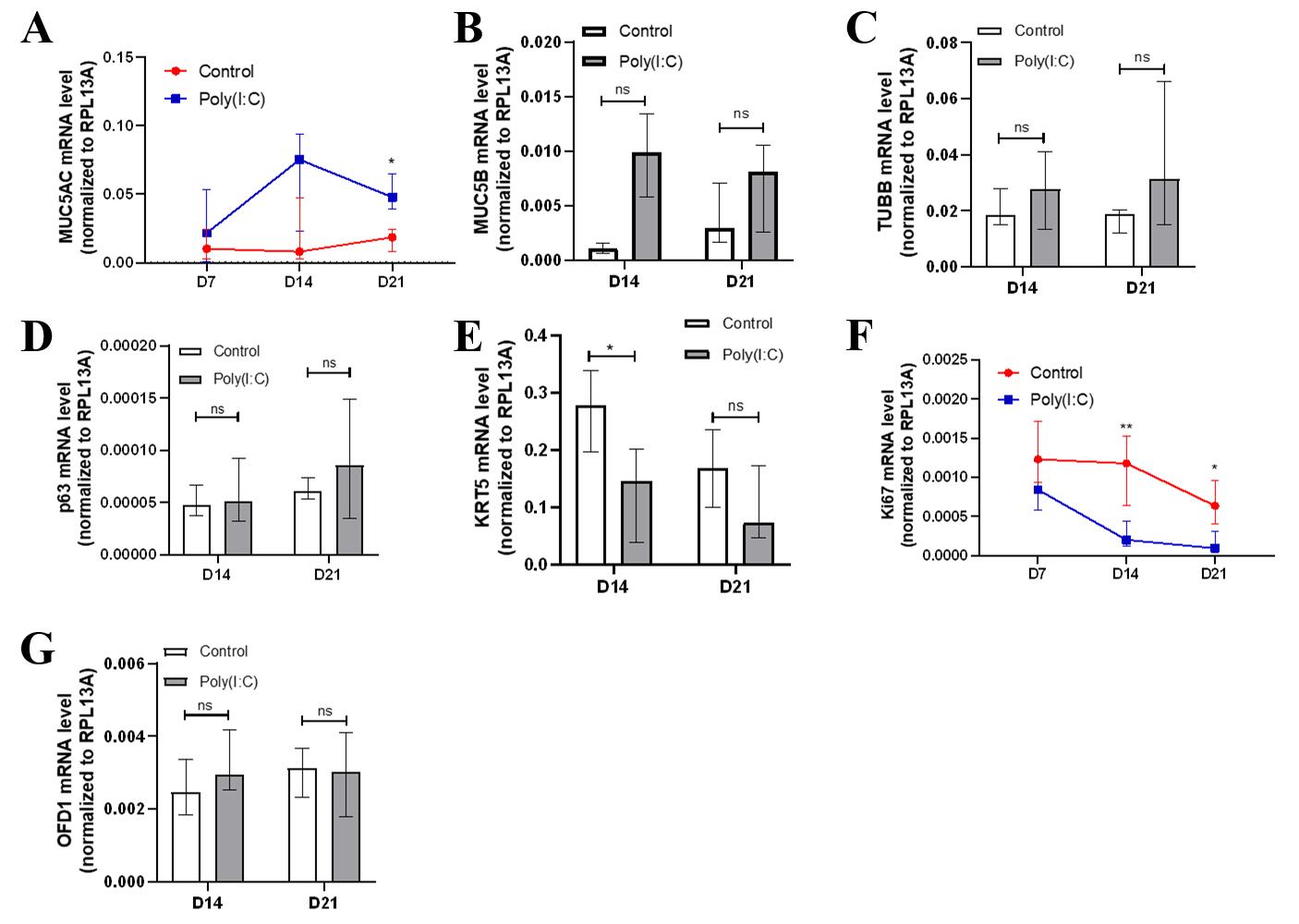


**Supplementary Figure 2.** (**A-B**) mRNA level of goblet cell markers *MUC5AC* and *MUC5B*. (**C, G**) mRNA level of cilia-associated markers *TUBB* and *OFD1*. (**D-F**) mRNA level of stem and proliferation markers *p63*, *KRT5* and *Ki-67*. Analysis was performed using Mann-Whitney test. Plot: median with IQR. (P<0.05 are presented as ‘*’. P < 0.01 are presented as ‘**’. ns: not significant. ALI – D7, n=3; D14, n = 6; D21, n=9.)


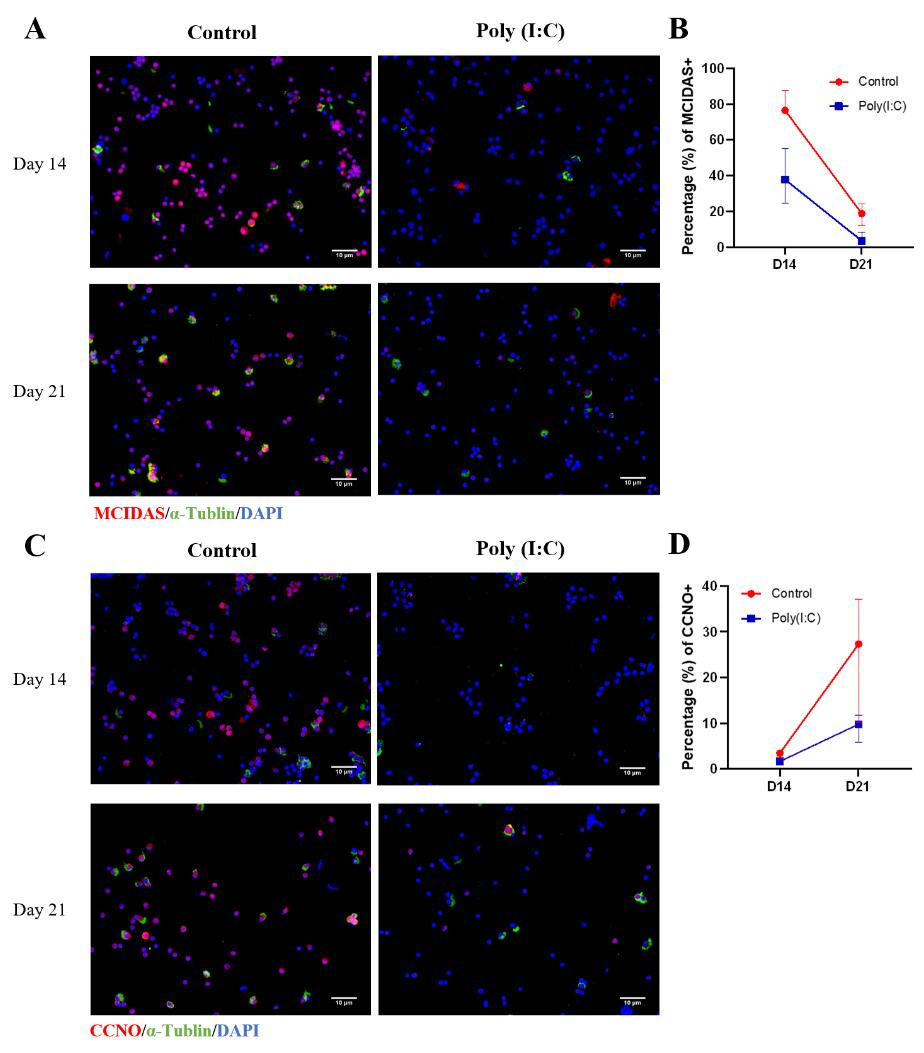


**Supplementary Figure 3.** (**A**) Representative cytospin IF images of ciliogenesis marker MCIDAS in differentiating hNECs at ALI – D14 and D21 (×200 magnification, scale bar = 10 μm). (**B**) Percentage of MCIDAS nuclear positive staining in total cells (counted in ×200 magnification, ALI - D14, n = 3; D21, n=3.) (**C**) Representative cytospin IF images of ciliogenesis marker CCNO in differentiating hNECs at ALI – D14 and D21 (×200 magnification, scale bar = 10 μm). (**D**) Percentage of CCNO nuclear positive staining in total cells (counted in ×200 magnification, ALI - D14, n = 3; D21, n=3.). Data was analysed using Mann-Whitney test. Plot: median with IQR.


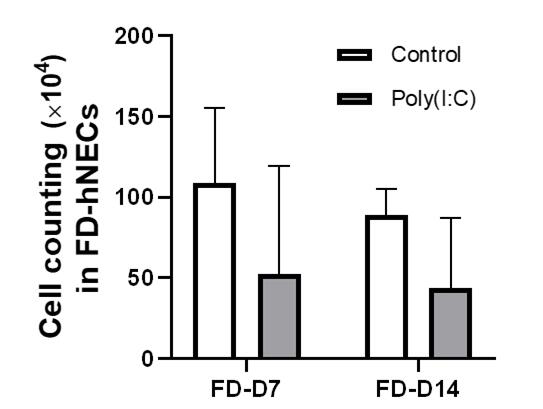


**Supplementary Figure 4.** Live cell count of fully differentiated hNECs treated by Poly(I:C) in ALI culture for 7 days and 14 days. (FD: fully differentiated). Data was analysed using Mann-Whitney test. Plot: median with IQR.


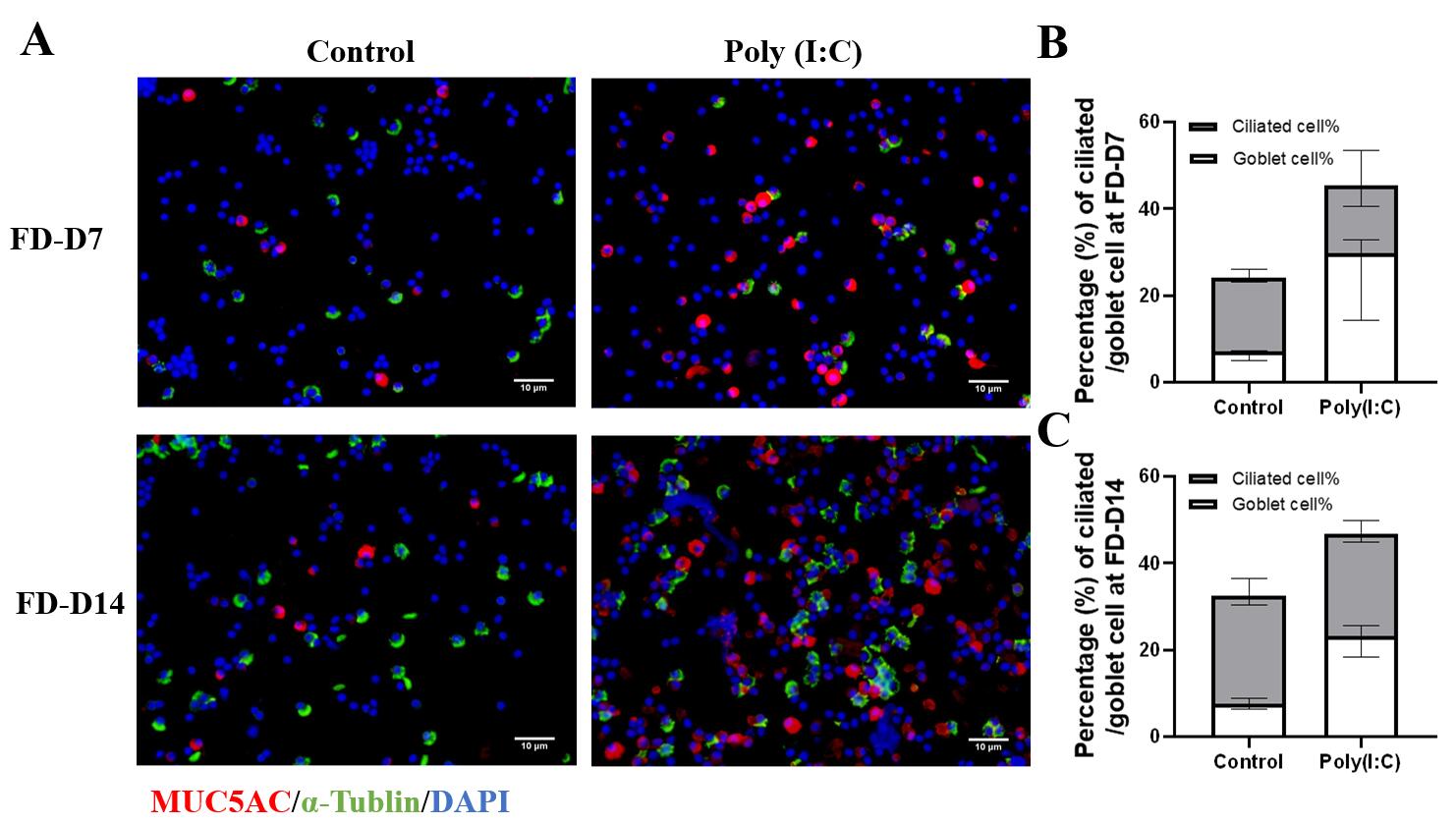


**Supplementary Figure 5.** Representative pictures of MUC5AC (red) and α-Tubulin (green)’s cytospin IF staining in fully differentiated hNECs post Poly(I:C) treatment for 7 days and 14 days. Data showed the percentages of goblet and ciliated cell counted in cytospin (counted in ×200 magnification, n=3). (FD: fully differentiated). Plot: median with IQR.


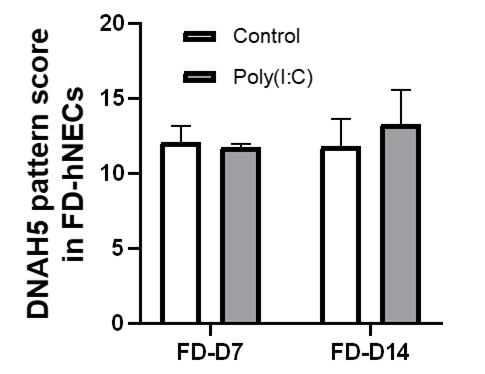


**Supplementary Figure 6.** DNAH5 pattern score in fully differentiated hNECs post Poly(I:C) treatment for 7 days and 14 days. (FD: fully differentiated. ALI - FD-D7: n=3; FD-D14: n=3). Plot: median with IQR.


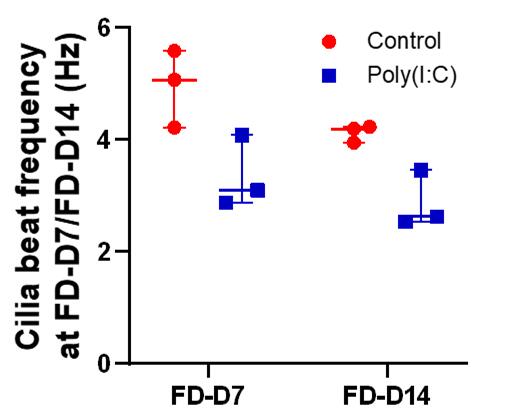


**Supplementary Figure 7.** CBF in fully differentiated hNECs post Poly(I:C) treatment for 7 days and 14 days. (FD: fully differentiated. ALI - FD-D7: n=3; FD-D14: n=3). Data was analysed using Mann-Whitney test. Plot: median with IQR.


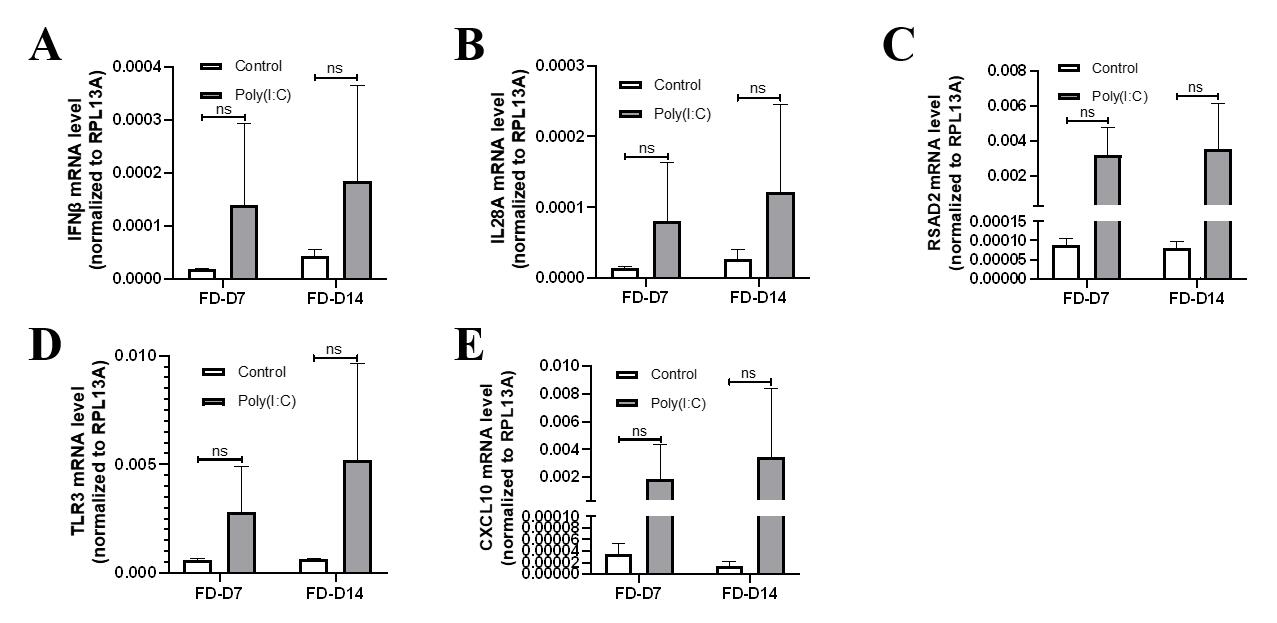


**Supplementary Figure 8.** (**A-E**) mRNA level of antiviral response markers in fully differentiated hNECs post Poly(I:C) treatment for 7 days and 14 days. (FD: fully differentiated. ns: not significant. ALI - FD-D7: n=3; FD-D14: n=3). Data was analysed using Mann-Whitney test. Plot: median with IQR.


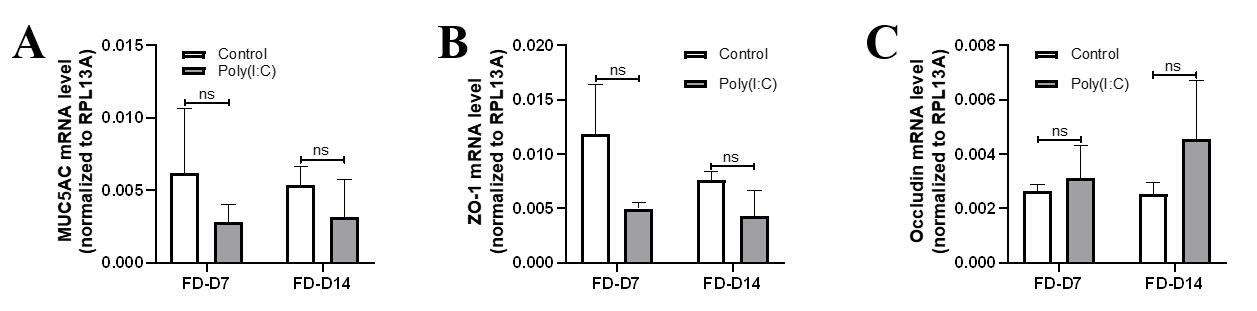


**Supplementary Figure 9.** (**A-C**) mRNA level of *MUC5AC* and TJ markers (*ZO-1* and *Occludin*) in fully differentiated hNECs post Poly(I:C) treatment for 7 days and 14 days. (FD: fully differentiated. ns: not significant. ALI - FD-D7: n=3; FD-D14: n=3). Data was analysed using Mann-Whitney test. Plot: median with IQR.


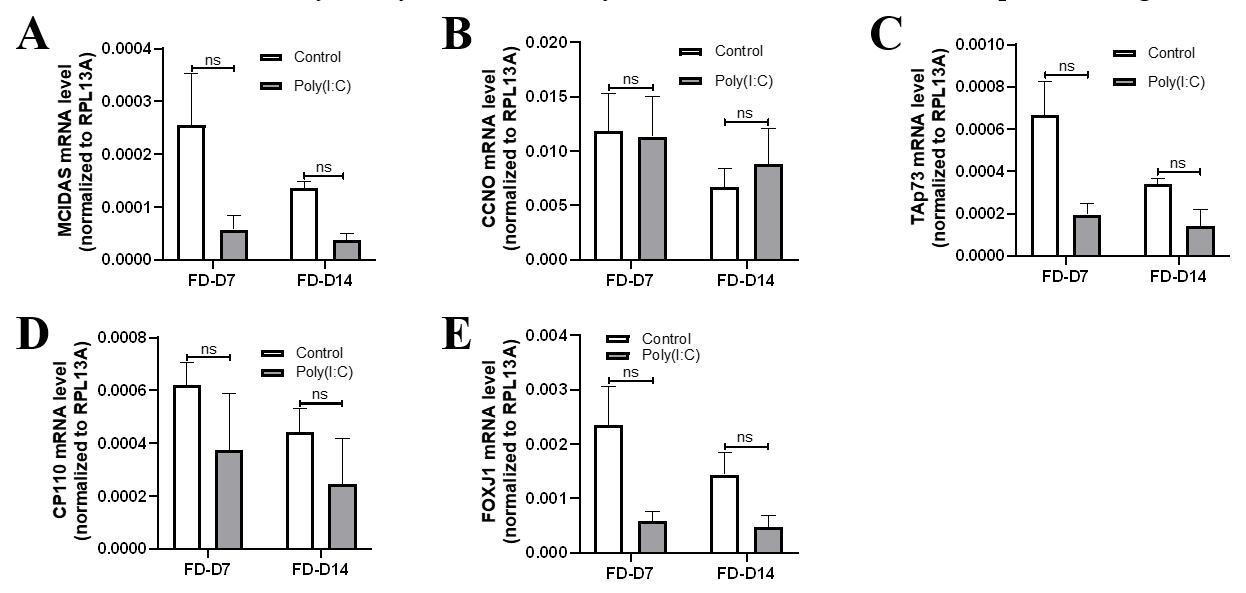


**Supplementary Figure 10.** (**A-E**) mRNA level ciliogenesis markers in fully differentiated hNECs post Poly(I:C) treatment for 7 days and 14 days. (FD: fully differentiated. ns: not significant. ALI - FD-D7: n=3; FD-D14: n=3). Data was analysed using Mann-Whitney test. Plot: median with IQR.


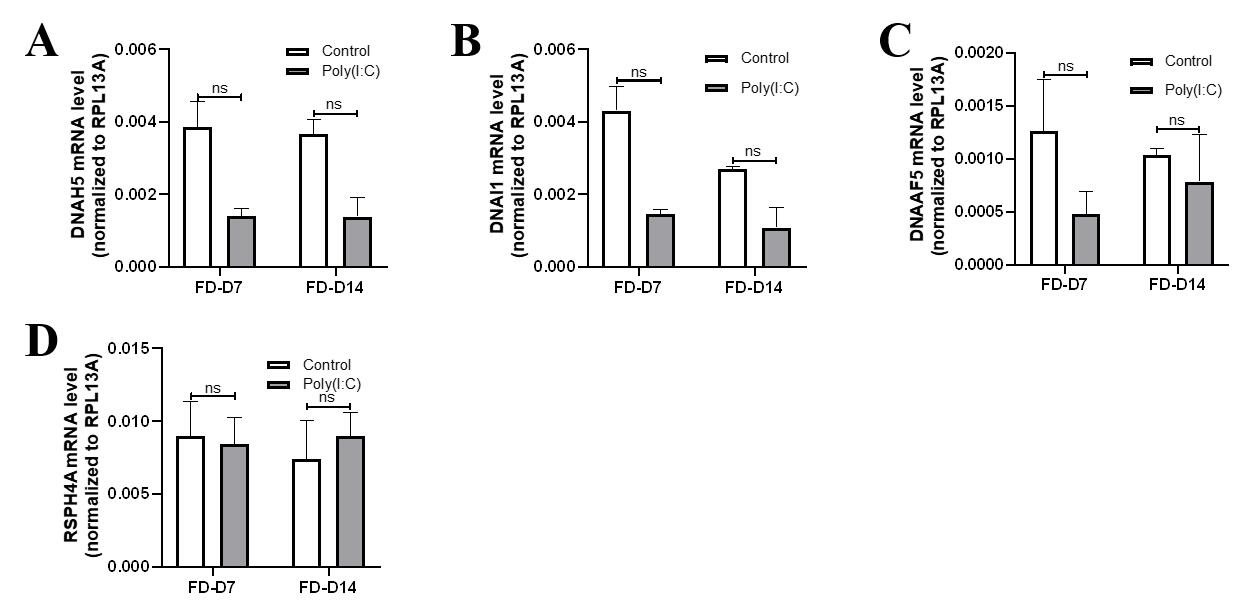


**Supplementary Figure 11.** (**A-D**) mRNA level cilium assembly markers in fully differentiated hNECs post Poly(I:C) treatment for 7 days and 14 days. (FD: fully differentiated. ns: not significant. ALI - FD-D7: n=3; FD-D14: n=3). Data was analysed using Mann-Whitney test. Plot: median with IQR.

## Supplementary Tables

**Supplementary Table 1.** Donor information of hNECs sources

| **Sample ID** | **Age** | **Gender** | **Diagnosis** | **Smoking history** | **Application** |
| --- | --- | --- | --- | --- | --- |
| V1 | 24 | M | SD | N | Virus infection |
| V2 | 28 | M | SD | N | Virus infection |
| V3 | 21 | M | SD | N | Virus infection |
| V4 | 24 | M | SD | N | Virus infection |
| V5 | 69 | M | SD | N | Virus infection |
| V6 | 35 | M | CRS | N | Virus infection |
| V7 | 32 | M | SD | N | Virus infection |
| V8 | 54 | M | SD | N | Virus infection |
| P1 | 34 | M | AR | Ex | Poly(I:C) treatment |
| P2 | 24 | M | SD | N | Poly(I:C) treatment |
| P3 | 26 | M | SD | N | Poly(I:C) treatment |
| P4 | 37 | M | CRS | N | Poly(I:C) treatment |
| P5 | 21 | M | AS | N | Poly(I:C) treatment |
| P6 | 35 | M | CRS | N | Poly(I:C) treatment |
| P7 | 52 | F | CRS | N | Poly(I:C) treatment |
| P8 | 54 | M | SD | N | Poly(I:C) treatment |
| P9 | 50 | F | CRS | N | Poly(I:C) treatment |
| P10 | 25 | F | AR | N | Poly(I:C) treatment |

Abbreviations: SD, septal deviation; CRS, chronic sinusitis; AS, acute sinusitis; AR, allergic rhinitis; Ex, Ex-smoker

**Supplementary Table 2.** Primer sequences used in RT-qPCR

| **Genes** | **Primer sequences** FW | **Primer sequences** RV |
| --- | --- | --- |
| *PGK1* | 5′-CTCAACAACATGGAGATTGG-3′ | 5′-CTTTGGACATTAGGTCTTTGAC-3′ |
| *RPL13A* | 5′-GTCTGAAGCCTACAAGAAAG-3′ | 5′-TGTCAATTTTCTTCTCCACG-3′ |
| *TLR3* | 5′-AGATTCAAGGTACATCATGC-3′ | 5′-CAATTTATGACGAAAGGCAC-3′ |
| *CXCL10* | 5′-AAAGCAGTTAGCAAGGAAAG-3′ | 5′-TCATTGGTCACCTTTTAGTG-3′ |
| *IFNB* | 5′-ATTCTAACTGCAACCTTTCG-3′ | 5′-GTTGTAGCTCATGGAAAGAG-3′ |
| *IL28A* | 5′-ACATAGCCCAGTTCAAGTC-3′ | 5′-GACTCTTCTAAGGCATCTTTG-3′ |
| *RSAD2* | 5′-GCTCTAAGAGAAGCAGAAAG-3′ | 5′-CATCTTCTGGTTAGATTCAGG-3′ |
| *FOXJ1* | 5′-GTGAAGCCTCCCTACTC-3′ | 5′-AATTCTGCCAGGTGGG-3′ |
| *CP110* | 5′-CTTAGTAGACAAGGAACCCC-3′ | 5′-CCGCTTTCTTTGGATTTTTC-3′ |
| *MCIDAS* | 5′-GGTGGATGATCTCATTTC-3′ | 5′-GAATGGTGATATGTCGCAAG-3′ |
| *CCNO* | 5′-ACAGTACTTCCTTGACTCAC-3′ | 5′-GGACTAAAAGGAAACGAAGG-3′ |
| *OFD1* | 5′-AAGCACCAAGAGATTGAAAC-3′ | 5′-CTTTATATTCTGCTCCTGTCTC-3′ |
| *DNAH5* | 5′-ACTGATGCAACTAATGAAGC-3′ | 5′-AGTGTAGGAATAGCATCCATC-3′ |
| *DNAI1* | 5′-TCAGTGGGAGATCTATGATG-3′ | 5′-ACTCCATAGATATCAGCTTC-3′ |
| *DNAAF5* | 5′-ATCCTGAACTCTTAAAACGC-3′ | 5′-GACACTGCTCTGATAGTAGG-3′ |
| *Tap73* | 5′-AAACTGCATCGAGTATTT-3′ | 5′-CAGGTCCTCAATGGTCAG-3′ |
| *TUBB* | 5′-CTTTGTATTTGGTCAGTCTGG-3′ | 5′-ATCTTGCTGATAAGGAGAGTG-3′ |
| *RSPH4A* | 5′-TTTGACACCAATCTCTGAAG-3′ | 5′-TTGTGGAATGAGATTTGAGG-3′ |
| *MUC5AC* | 5′-AATGGTGGAGATTTTGACAC-3′ | 5′-TTCTTGTTCAGGCAAATCAG-3′ |
| *MUC5B* | 5′-TACGTTCTGTCCAAGAAATG-3′ | 5′-TAGATGGAGTTGAGGAACAC-3′ |
| *ZO-1* | 5′- TTGTCTTCAAAAACTCCCAC -3′ | 5′- GACTCACAGGAATAGCTTTAG -3′ |
| *Occludin* | 5′- GGACTGGATCAGGGAATATC -3′ | 5′- ATTCTTTATCCAAACGGGAG -3′ |
| *Ki67* | 5′-GACAGAGGTTCCTAAGAGAG-3′ | 5′-AACAATCAGATTTGCTTCCG-3′ |
| *p63* | 5′-CAGCCTATATGTTCAGTTCAG-3′ | 5′-CAGTCCATGCTAATCTCAATC-3′ |
| *KRT5* | 5′-TGGAAGACTTCAAGAACAAG-3′ | 5′-ATGTAGGCAGCATCTACATC-3′ |
